# The finely defined shift work schedule of dung beetles and their eye morphology

**DOI:** 10.1002/ece3.8264

**Published:** 2021-10-27

**Authors:** Claudia Tocco, Marie Dacke, Marcus Byrne

**Affiliations:** ^1^ Lund Vision Group Department of Biology Lund University Lund Sweden; ^2^ School of Animal, Plant and Environmental Sciences University of the Witwatersrand Wits, Johannesburg South Africa

**Keywords:** body size, cornea, diel activity period, morphological light adaptations, ocular canthus, Scarabaeinae

## Abstract

In nature, nothing is wasted, not even waste. Dung, composed of metabolic trash and leftovers of food, is a high‐quality resource and the object of fierce competition. Over 800 dung beetle species (Scarabaeinae) compete in the South African dung habitat and more than 100 species can colonize a single dung pat. To coexist in the same space, using the same food, beetles divide the day between them. However, detailed diel activity periods and associated morphological adaptations have been largely overlooked in these dung‐loving insects. To address this, we used a high‐frequency trapping design to establish the diel activity period of 44 dung beetle species in their South Africa communities. This allowed us to conclude that the dung beetles show a highly refined temporal partitioning strategy, with differences in peak of activity even within the diurnal, crepuscular, and nocturnal guilds, independent of nesting behavior and taxonomic classification. We further analyzed differences in eye and body size of our 44 model species and describe their variability in external eye morphology. In general, nocturnal species are bigger than crepuscular and diurnal species, and as expected, the absolute and relative eye size is greatest in nocturnal species, followed by crepuscular and then diurnal species. A more surprising finding was that corneal structure (smooth or facetted) is influenced by the activity period of the species, appearing flat in the nocturnal species and highly curved in the diurnal species. The role of the canthus—a cuticular structure that partially or completely divides the dung beetle eye into dorsal and ventral parts—remains a mystery, but the large number of species investigated in this study nevertheless allowed us to reject any correlation between its presence and the nesting behavior or time of activity of the beetles.

## Graphical abstract

Dung is the site of fierce competition. To coexist in exactly the same space, using precisely the same food, over 40 species of dung beetles are shown in our paper to finely divide the day between them. This is driven by changes in light, which in turn reflect in morphological adaptations among these species which in many cases override their taxonomic heritage.

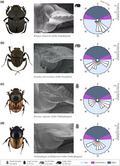

## 1 INTRODUCTION

The ecological niche of a species can be imagined as a multidimensional space where the different axes represent the conditions that allow the individuals of that species to survive and reproduce, such as habitat, food, temperature, time, competition, and predation (Kearney, 2006; Tsunoda et al., 2020). According to current niche theories, species can coexist only if their niches are not identical (Kearney, 2006; Leibold, 1995). Competitive interactions occur whenever an individual reduces the availability of a shared resource to another individual or negatively affects the ability of another individual to use the shared resource (Carothers et al., 1984). Intra‐ and interspecific competition for a resource often leads to spatial and temporal niche partitioning, boosting diversity, and structuring natural populations and communities (Tilman, 1982). For example, in periods of high energetic requirements, intraspecific competition in brown trout *Salmo trutta* results in sequential use of shared foraging areas based on their social rank, with dominant individuals feeding mainly at dusk and early night (the most beneficial times) and second‐ranking fish become mainly diurnal (Alanärä et al., 2001). In the Balkan Mountains, interspecific competition among mammalian carnivores (golden jackal, European badger, red fox, European wildcat, and stone marten) also results in spatial and temporal (both diel and seasonal) partitioning; the more similar the trophic niche the greater the partitioning (Tsunoda et al., 2020). In hot rocky deserts, temporal partitioning allows the nocturnal spiny mouse *Acomys cahirinus* and the diurnal *A. russatus* to coexist under different environmental challenges; high physiological costs for the diurnal species during the hot summer, while an elevated energetic cost of thermoregulation is compulsory for the nocturnal species in winter (Kronfeld‐Schor et al., 2001). However, it is important to note that the mechanism of niche differentiation for species coexistence and increased diversity has been criticized by some authors (Connell, 1980), who suggest that adaptations already possessed by the species are causally linked to their successful coexistence. Alternatively, harsh conditions, which constrain a species’ numbers, may also allow coexistence.

Dung is a scattered food source that supports about 7000 dung beetle (Coleoptera: Scarabaeinae) species worldwide (Schoolmeesters, 2021); over 800 dung beetle species compete in the Southern Africa dung habitat and more than 100 species can colonize a single dung pat (Bernon, 1981). Therefore, coprophagous beetle assemblages provide us with a clear example of closely related insects that coexist in precisely the same habitat, presumably by partitioning the available resources in some way. Beetles avoid competition by niche differentiation along several ecological axes, including their trophic preferences (Frank et al., 2017), and association with different vegetation and soil types, altitude, or climate (Chamberlain et al., 2015; Davis & Scholtz, 2020; Tocco et al., 2020), as well as alternative feeding and nesting strategies. These include dwelling (feeding and breeding within the dung pat or in the soil–dung interface), tunneling (storing dung for feeding and breeding in tunnels directly underneath the dung pat), or rolling (balls of dung for feeding or breeding are rolled away from the dung pad and buried) (Hanski & Cambefort, 1991). Other important niche differentiations occur along temporal axes, such as colonizing dung at different stages of decomposition (Lee & Wall, 2006), a strong seasonality (Kamiński et al., 2015; Latha, 2019; Lobo & Cuesta, 2021), or a marked diel activity period (Caveney et al., 1995; Feer & Pincebourde, 2005; Kamiński et al., 2015; Tocco et al., 2019). In this case, the availability of light during the period of activity is expected to influence eye morphology, depending on the time when the species is active.

Dung beetles have compound eyes, divided into a dorsal and a ventral part which is often separated by the ocular canthus (a cuticular ridge) in some species, or it can be so reduced that the eye appears as a single structure in other species. The role of this cuticular structure is not clear, but has been suggested to protect the eye from abrasion in the soil (Scholtz & Davies, 2009). Each single element of the compound eye, termed an ommatidium, can usually be observed from the surface of the eye as a clearly defined facet. Some nocturnal and crepuscular dung beetle species, however, have a smooth cornea, as a result of which the ommatidia cannot be detected on the surface of the eye (Byrne & Dacke, 2011; Caveney & McIntyre, 1981; Dacke et al., 2003; Tocco et al., 2019). As information on diel activity period and external eye structure for most dung beetle species is currently lacking, it has been hard to draw any firm conclusions regarding the adaptive value of these highly visible external morphological characteristics of the beetle eye or its relationship with body size within the group. In this study, we describe the diel activity period of 44 dung beetle species occurring in Southern Africa. Together with characterizations of external eye morphologies (presence or absence of a complete canthus, faceted or smooth cornea surface, and absolute and relative eye size) allows us to establish possible relationships between eye morphology and taxonomic classification at tribe level, nesting behavior, and time of activity.

## 2 MATERIALS AND METHODS

### 2.1 Collection of specimens

Dung beetle species belonging to the subfamily Scarabaeinae Latreille were collected in South Africa at Stonehenge game farm, North West province (26°28′14.0″S 24°20′30.8″E), Pullen nature reserve, Mpumalanga province (25°34′01.6″S 31°10′42.5″E), and near the town of Bela Bela, Limpopo Province (24°46′04.0″S 27°56′37.3″E). All three sampling locations are in the Savanna biome. More specifically, Stonehenge game farm is in the Eastern Kalahari Bushveld Bioregion, Mafikeng Bushveld (SVk1), Pullen nature reserve is in the Lowveld Bioregion, Malelane Mountain Bushveld (SVl11), and the Bela Bela site is in the Central Bushveld Bioregion, Central Sandy Bushveld (SVcb12) (Mucina & Rutherford, 2006). A total of ten 24 h sampling sessions were performed between the months of November and February in the years 2016 to 2020. The sampling took place during the rainy season in mid‐to‐late summer to accommodate the seasonal activity peak of adult dung beetles in the three sampling locations. Sampling was conducted on warm, clear days and nights, shortly after rain (see Supporting Information).

### 2.2 Diel activity period

Between five and ten dung baited pitfall traps of the flat‐bait trap type (Tocco et al., 2017), baited with 200 g of fresh cow dung, were placed at each site separated by a minimum distance of 50 m. The traps were half filled with a 20% aqueous solution of ethylene glycol to prevent beetle escape and preserve specimens for morphological identification. Once collected, specimens were preserved in 75% ethanol for identification in the laboratory, using dichotomous keys (Deschodt et al., 2015; D'Orbigny, 1913; Ferreira, 1961; Janssens, 1953; Montreuil, 2015; Paschalidis, 1974). To precisely determine the diel activity period of the trapped beetle species, the traps were periodically emptied and re‐baited with fresh dung at set intervals during each 24 h sampling session (18 times per 24 h at Stonehenge and Bela Bela sites, and 10 times in the Pullen nature reserve [Supporting Information]). At Stonehenge and Bela Bela, the traps were emptied and re‐baited every 2 h during daylight (between sunrise and sunset, i.e., when the geometric center of the sun is ≥0° above the horizon) and night time (when the geometric center of the sun is more than 18° below the horizon), and every 30 min during dusk and dawn (when the geometric center of the sun is between 0° and 18° below the horizon) (Tocco et al., 2019). At Pullen nature reserve, the traps were emptied and re‐baited approximately every 2.5 h during daylight, every 3 h at night, and every 1 h and 20 min during dusk and dawn.

Forty‐three dung beetle species, each with a total abundance greater than 100 individuals were classified: (1) ecologically as diurnal, nocturnal, or crepuscular based on when they reached their activity peak; (2) morphologically based on the external structure of the eye; and (3) behaviorally as rollers, tunnelers, or dwellers based on their known nesting and dung‐handling behaviors (Hanski & Cambefort, 1991). Despite a total abundance of only 23 individuals, the crepuscular *Onitis uncinatus* Klug was also included in the study to add a further combination of ecological, morphological, and behavioral features that otherwise would have been missed; the activity patterns of *O. uncinatus* recorded in our samples were in agreement with that previously reported by Caveney et al. (1995). This gave a total of 44 selected species all of which fly to forage for fresh dung. Voucher material is deposited at the Life Sciences Museum, University of the Witwatersrand, Johannesburg, South Africa.

### 2.3 Comparisons of eye morphology and eye size between species

**FIGURE 1:**
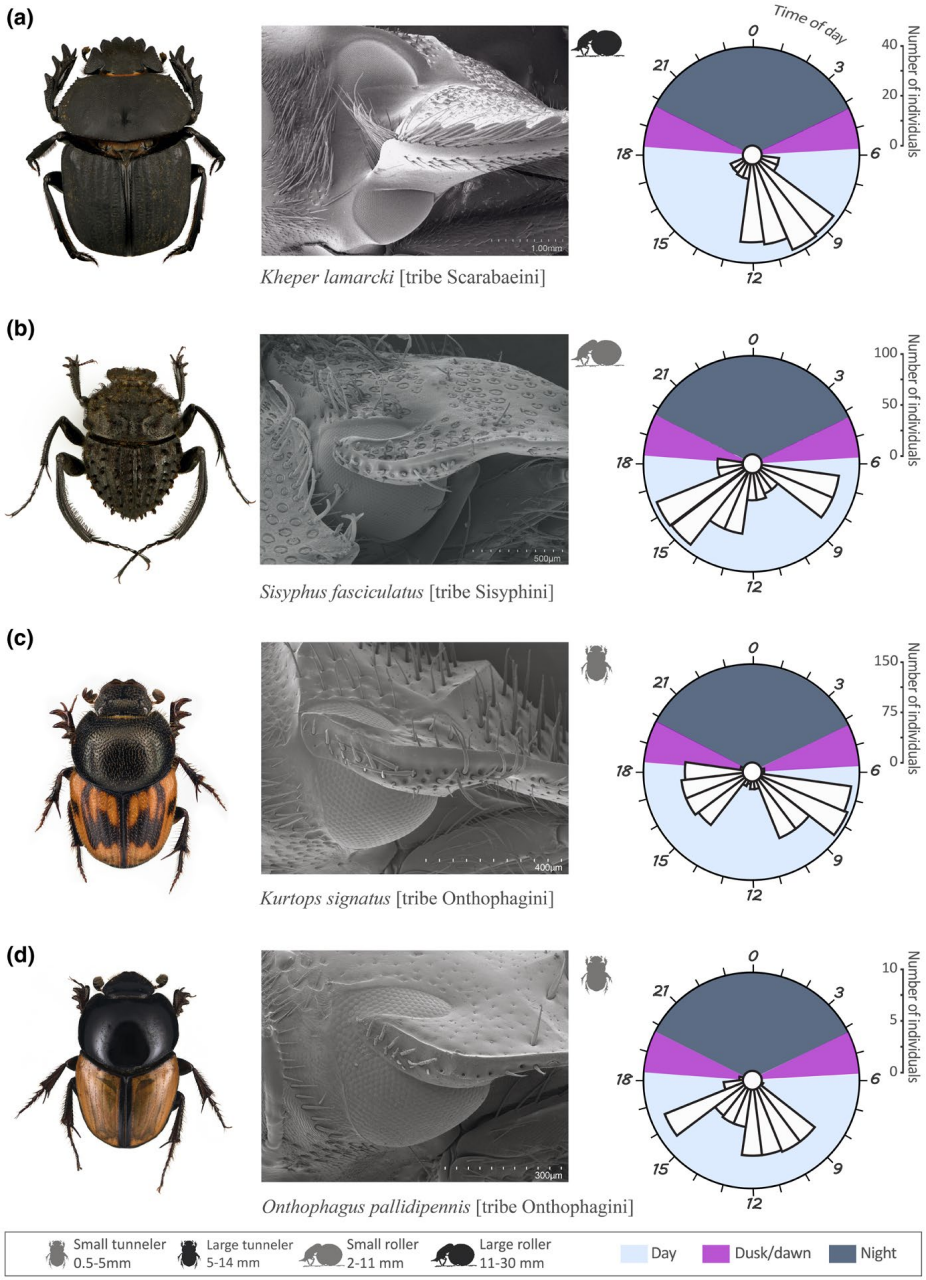
For the diurnal model species (a) *Kheper lamarcki*, (b) *Sisyphus fasciculatus*, (c) *Kurtops signatus*, and (d) *Onthophagus pallidipennis*, we show (1) the *habitus* (general appearance of the species in dorsal view, body size of the species is given in Table 1, 2), (2) a SEM of the right eye to depict the eye type, and (3) a circular plot of the diel activity period, depicted as the mean abundance of a species in the different sampling times over a period of 24 h

**FIGURE 2:**
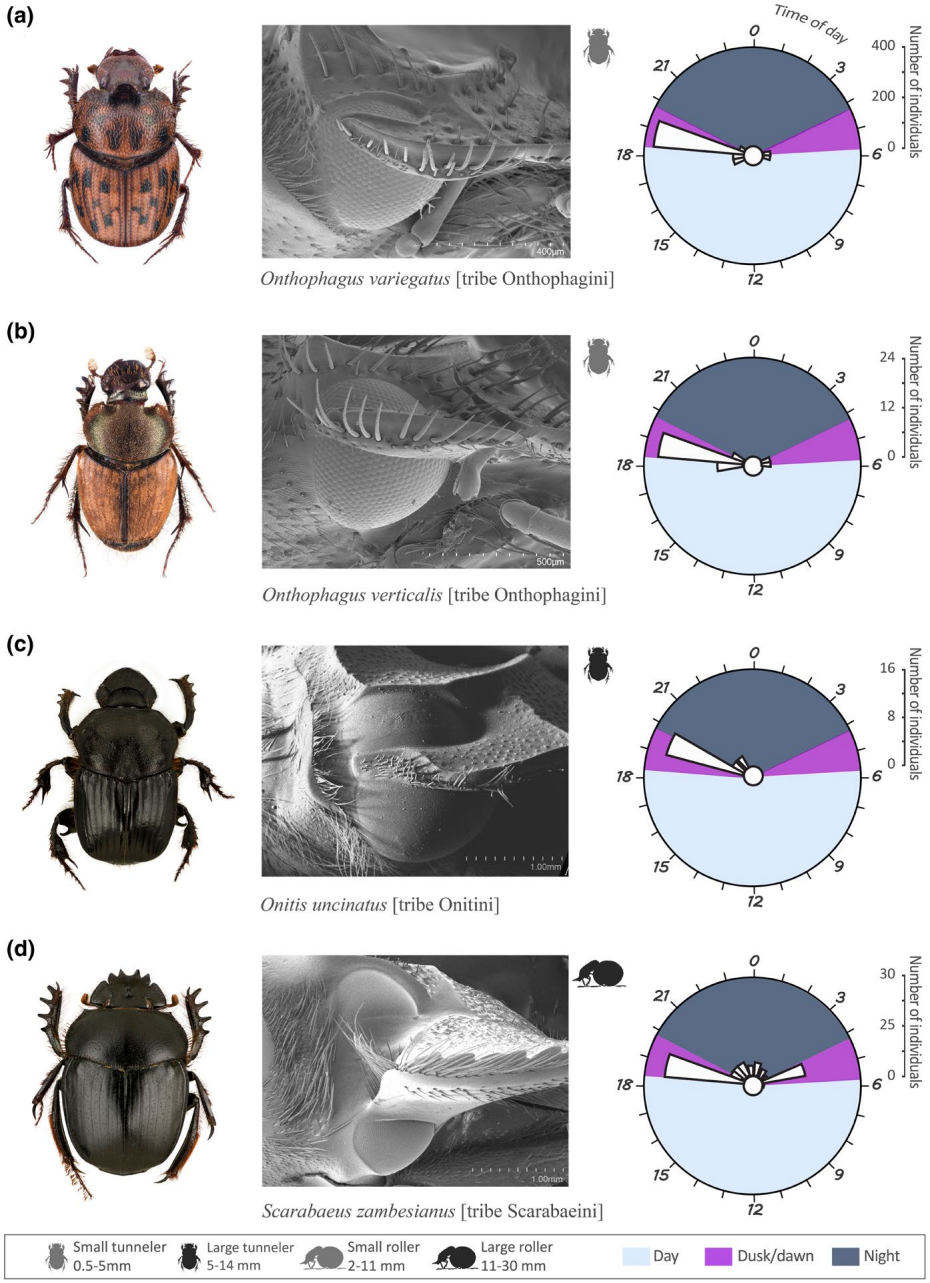
For the crepuscular model species (a) *Onthophagus variegatus*, (b) *Onthophagus verticalis*, (c) *Onitis uncinatus*, and (d) *Scarabaeus zambesianus*, we show (1) the *habitus* (general appearance of the species in dorsal view, body size of the species is given in Table 2), (2) a SEM of the right eye to depict the eye type, and (3) a circular plot of the diel activity period, depicted as the mean of abundance of a species in the different sampling times over a period of 24 h

**FIGURE 3:**
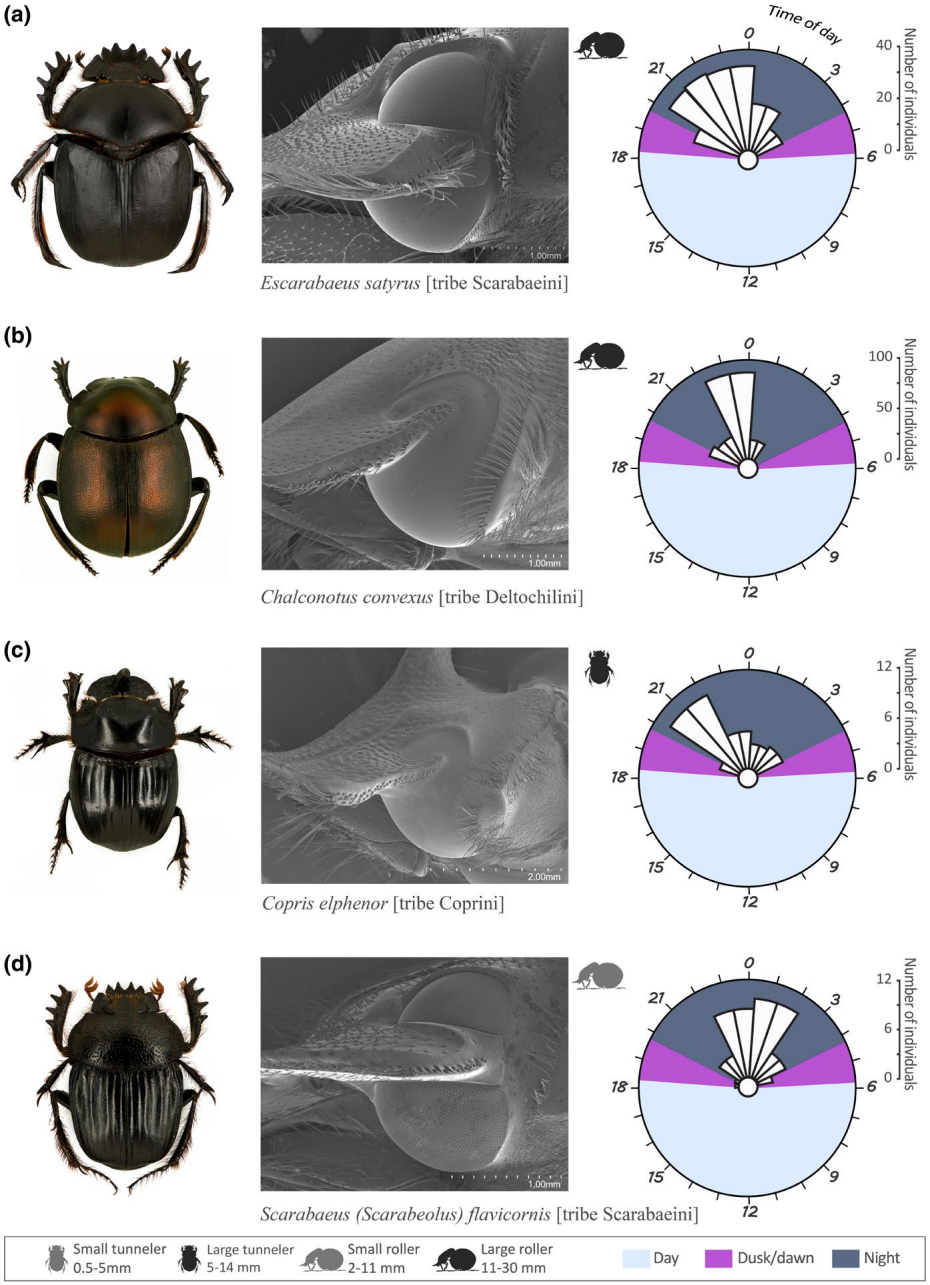
For the nocturnal model species (a) *Escarabaeus satyrus*, (b) *Chalconotus convexus*, (c) *Copris elphenor*, and (d) *Scarabaeus* (*Scarabaeolus*) *flavicornis*, we show, (1) the *habitus* (general appearance of the species in dorsal view, body size of the species is given in Table 2), (2) a SEM of the right eye to depict the eye type, and (3) a circular plot of the diel activity period, depicted as the mean of abundance of a species in the different sampling times over a period of 24 h

The variability in external eye structure (faceted/smooth cornea and presence/absence of a canthus) of the 44 dung beetle species was studied using light microscopy and scanning electron microscopy (SEM). Individuals used for the scanning electron microscopy were cleaned with a mixture of water and detergent, rinsed in 75% ethanol, air dried, and sputter coated with gold (65 s, 20 mA). For each of the 12 combinations of ecological (diurnal/crepuscular/nocturnal), morphological (presence/absence of a complete canthus and faceted/smooth cornea), and behavioral (roller/tunneler) features found, we randomly selected a model species for illustration (Figures 1, 2, 3). For example, *Kheper lamarcki* (Mac Leay) (Figure 1a) was used as a representative of a *diurnal* and *roller* species with an eye *with a complete canthus and a faceted cornea*. For the 12 model species, we noted (1) the diel activity period, depicted as the mean of abundance of the species in the different sampling times over a period of 24 h, plotted with *ggplot2* version 3.2.0, (2) a SEM of the right eye to depict the eye type, and (3) the *habitus* (general appearance of the species in dorsal view).

To investigate associations between eye size and diel activity period, taking into account the effect of body size, we also measured the relative eye area of the 12 model species selected as above. An additional species was sought for each of the 12 combinations to increase the strength of our analysis; however, only seven additional species from the total collected were suitable, giving a total of 19 species. To measure the area of the curved eye surface, the heads of eight to ten individuals of each species (total number of individuals = 170) were removed and the right eye was covered with a thin layer of transparent nail polish. After about 20 min, the nail polish was peeled off producing a clear impression of the eye surface which was then cut to be mounted flat on a microscope slide. Images of the flattened impression of the eyes were taken with a stereo microscope (Zeiss, Discovery V12) and the absolute area was measured using ImageJ ver. 1.50i (Rasband, 2012). For the species with a complete canthus (dividing the eye in two), absolute eye area was calculated from the sum of the dorsal and ventral eye areas of the same individual. The maximum width of the pronotum in dorsal view of each specimen was measured using a digital caliper (Cocraft 0–150 mm) and used as a proxy for body size. This allowed us to calculate the relative eye area as the ratio between absolute eye area and body size.

To investigate differences in absolute and relative eye area (19 species) and body size (44 species) among diurnal, crepuscular, and nocturnal species, generalized linear mixed models (GLMMs) were fitted using the package lme4 (Bates et al., 2014). Absolute and relative eye area and body size were specified as dependent variables in the respective models. All fitted models accounted for fixed effects of diel activity period. Random effects of the tribe and nesting behavior were specified in the eye area models and body size model, respectively. Model selections were based on the conditional Akaike Information Criterion (AIC) using the package cAIC4 (Säfken et al., 2018). Shapiro–Wilk normality tests and Q–Q plots did not confirm the normality of errors, and the gamma distribution was thus specified in the models (Zuur et al., 2007). Similarly, the presence/absence of a complete ocular canthus among diurnal, crepuscular, and nocturnal species, and tunneler and rollers, was investigated by fitting a GLMM with binomial‐distributed errors, diel activity period and nesting behavior were specified as fixed effects, and tribe as a random effect. Post hoc interaction analysis was performed with the Package *Phia* (De Rosario‐Martinez, 2015), using the Chi‐squared test, and correcting the p‐values according to the Holm method, to calculate pairwise differences in absolute eye area, relative eye area, and body size between diel activity periods, and in the presence/absence of a complete canthus between diel activity periods and nesting behaviors. All analyses were run in R 3.6.1. (R Core Team, 2019).

## 3 RESULTS

A total of 17,756 dung beetles belonging to the subfamily Scarabaeinae were collected; at Stonehenge game farm (10,936 individuals of 52 species) and at Pullen nature reserve (6820 individuals of 35 species) (Supporting Information). In addition, 23 individuals of the species *Onitis uncinatus* were collected at our Bela Bela field site. Further analysis of activity patterns and morphological adaptations was limited to the 44 study species from eight tribes of dung beetles (Supporting Information).

### 3.1 Diel activity period of the species

#### 3.1.1 Diurnal species

Twenty‐seven of the 44 species included in this study were active only during the day (between sunrise and sunset, i.e., when the geometric center of the sun was ≥0° above the horizon), 12 with a single, well‐defined peak of activity, and 15 with dual peaks of activity (Tables 1 and 2). The unimodally active group contained three ball‐rolling species from the tribe Scarabaeini; *Scarabaeus* (*Scarabaeolus*) *carniphilus* Davis and Deschodt, *Kheper lamarcki* (Mac Leay) (Figure 1a), and *Pachylomera femoralis* Kirby, all reached their peak of activity midmorning, while the three tunneling species from the tribe Onthophagini *Onthophagus flavolimbatus* Klug, *Proagoderus sapphirinus* Fåhr, and *Kurtops quadraticeps* (Harold), and the roller *Allogymnopleurus splendidus* (Bertoloni) from tribe Gymnopleurini, were mainly active at noon (Table 1). The roller *Garreta unicolor* (Fahraeus) [tribe Gymnopleurini], the tunnelers *Afrodrepanus impressicollis* (Boheman) and *Liatongus militaris* (Laporte) [tribe Oniticellini], and *Onthophagus cribripennis* D'Orbigny and *Onthophagus sugillatus* Klug reached their peak of activity midafternoon (Table 1).

**TABLE 1:**
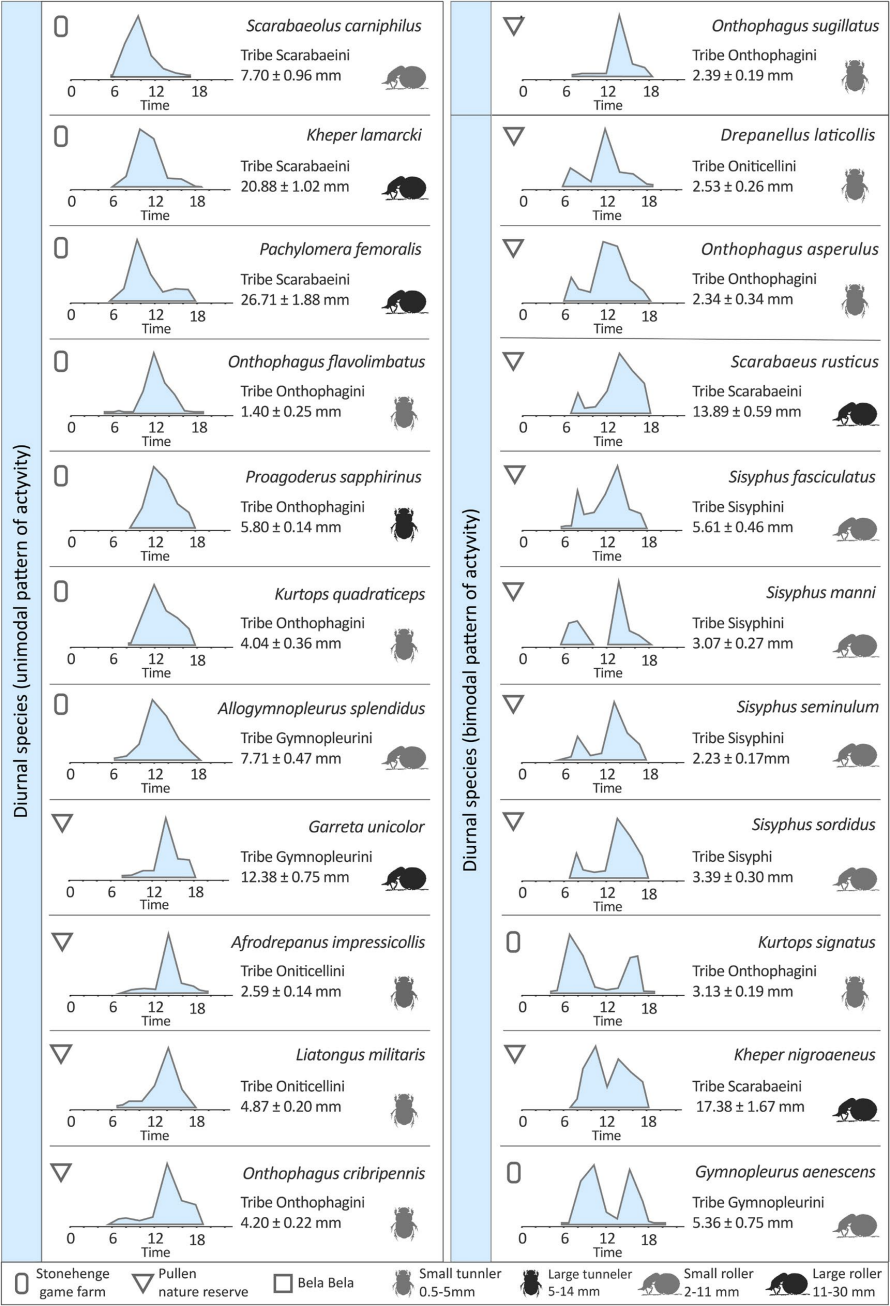
Activity period of 22 diurnal dung beetle species from two different collection sites (Stonehenge game farm and Pullen nature reserve)

Note

Beetles were periodically collected during several 24 h sampling occasions using dung‐baited pitfall traps. Activity period is shown as the proportion of each species’ total numbers sampled at a given time. For each species, tribe, body size (maximum width of the pronotum), and nesting behavior (roller or tunneler) are also given. Activity periods are shown as unimodal ranked from earliest to latest, followed by increasingly bimodal activity periods.

**TABLE 2:**
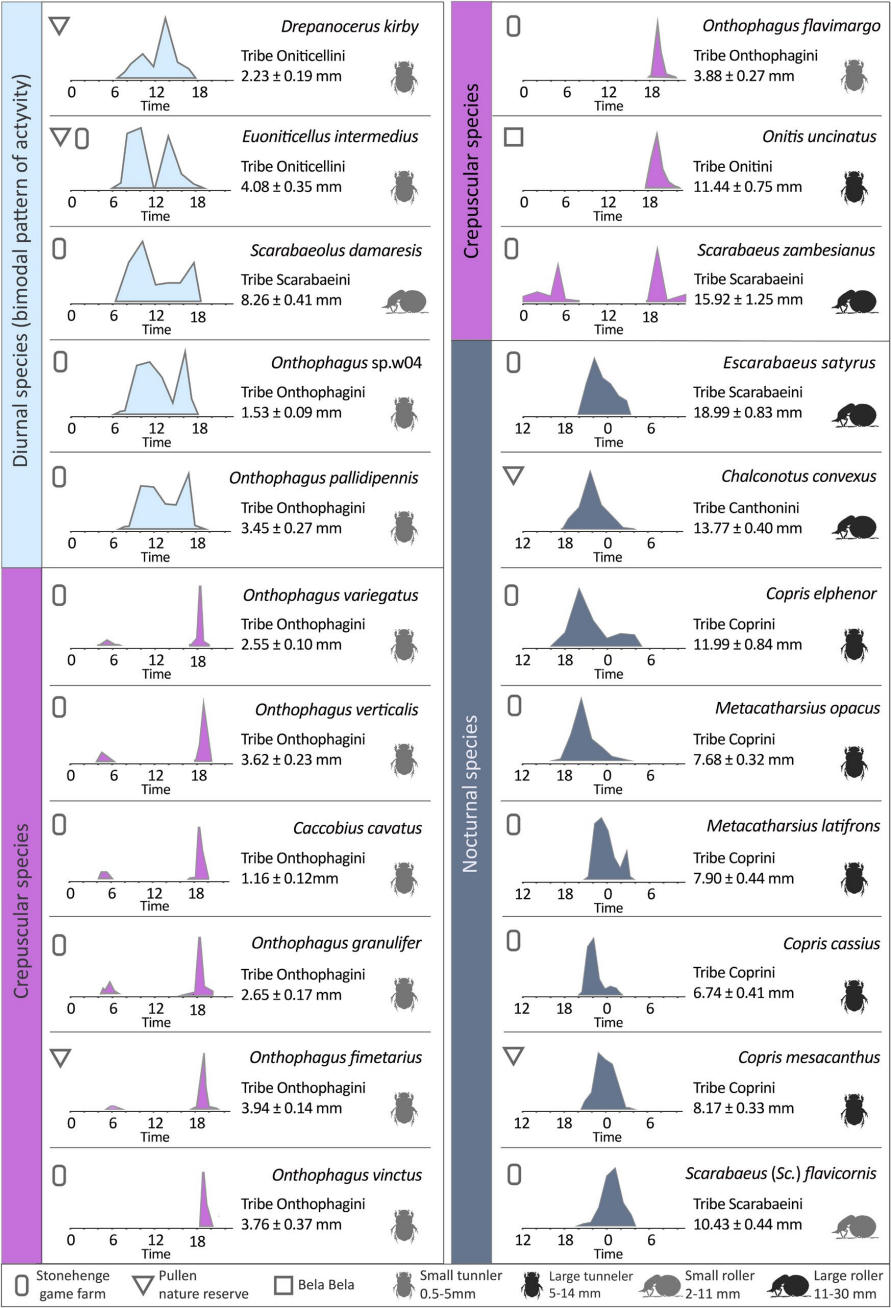
Activity period of five diurnal, nine crepuscular, and eight nocturnal dung beetle species from three different collection sites (Stonehenge game farm, Pullen nature reserve, and Bela Bela)

Note

Beetles were periodically collected during several 24 h sampling occasions using dung‐baited pitfall traps. Activity period is shown as the proportion of each species’ total numbers sampled at a given time. For each species, tribe, body size (maximum width of the pronotum), and nesting behavior (roller or tunneler) are also given.

Amongst the 15 day‐active species that showed bimodal activity, five different combinations were observed. Two tunnelers; *Drepanellus laticollis* (Fahraeus) [tribe Oniticellini] and *Onthophagus asperulus* D'Orbigny were primarily active in the early morning and around noon (Table 1). Five rollers: *Scarabaeus rusticus* (Boheman), *Sisyphus fasciculatus* Boheman (Figure 1b), *Sisyphus manni* Montreuil, *Sisyphus seminulum* Gerstaecker, 1871, and *Sisyphus sordidus* Boheman [tribe Sisyphini] were mainly active in the early morning and midafternoon (Table 1). The tunneler *Kurtops signatus* (Fahraeus) (Figure 1c) reached its peak of activity in the early morning and the late afternoon, and some individuals even occurred in the traps at dusk. The rollers *Kheper nigroaeneus* (Boheman) and *Gymnopleurus aenescens* Wiedemann [tribe Gymnopleurini] (Table 1), and the tunnelers *Drepanocerus Kirbyi* Kirby and *Euoniticellus intermedius* (Reiche) [tribe Oniticellini] were instead active midmorning and midafternoon (Table 2). Finally, the roller *Scarabaeus* (*Scarabaeolus*) *damarensis* Janssens and the tunnelers *Onthophagus pallidipennis* Fahraeus (Figure 1d) and *Onthophagus* sp.w04 were mainly active midmorning and late afternoon (Table 2).

#### 3.1.2 Crepuscular species

Nine of the 44 study species were crepuscular (i.e., with peak of activity at dusk and/or dawn). The tunnelers *Onthophagus variegatus* Fabricius (Figure 2a), *Onthophagus verticalis* Fahraeus (Figure 2b), *Caccobius cavatus* D'Orbigny [tribe Onithophagini], and *Onthophagus granulifer* Harold were exclusively crepuscular, with a marked peak in activity at the onset of dusk (Table 2). The tunneler *Onthophagus fimetarius* Roth was also active at dusk and dawn, but with 4 individuals of 156 still active just after dusk. The tunnelers *Onthophagus vinctus* Erichson appeared in the traps only at dusk, while few individuals of *Onthophagus flavimargo* D'Orbigny and *Onitis uncinatus* [tribe Onitini] (Figure 2c) (4 of a total of 122 and 6 of a total of 23 individuals, respectively) extending their activity into the early night (Table 2). The roller *Scarabaeus zambesianus* Péringuey (Figure 2d) was mainly active at dusk *and* dawn, and also extending its activity into the night (Figure 2c, Table 2).

#### 3.1.3 Nocturnal species

Eight of the 44 species were nocturnal. The two rollers *Escarabaeus satyrus* (Boheman) [tribe Scarabaeini] (Figure 3a) and *Chalconotus convexus* Boheman [tribe Deltochilini] (Figure 3b), and the five tunnelers *Copris elphenor* Felsche (Figure 3c), *Metacatharsius opacus* Waterhouse, *Metacatharsius latifrons* (Harold), *Copris cassius* Péringuey, and *Copris mesacanthus transvaalensis* Nguyen‐phung [tribe Coprini] became active at dusk. These five species were mostly active during the first half of the night with a peak of activity between the early night and midnight (Table 2). The roller *Scarabaeus* (*Scarabaeolus*) *flavicornis* (Boheman) (Figure 3d), however, did not reach its peak of activity until 1 h after midnight (Table 2).

### 3.2 Comparisons of external eye morphology between species

Among our sample of 44 study species, 19 species have their eye completely divided into two halves by a canthus. The GLMM and associated interaction test show that among this selection of species, the presence of a complete canthus was neither associated with nesting behavior (presence of complete canthus, roller–tunneler: value = 0.8824, Chisq = 0.123, *p* = .72) nor time of flight activity (complete canthus: *diurnal–nocturnal*; value = 0.11, Chisq = 0.21, *p* = .91). A less obvious trait, at least to the human eye, is the facetted or smooth cornea surface (Figure 2c), which is facetted in all 27 diurnal species. While only 2 of the 9 crepuscular species of this study had a smooth cornea (*S. zambesianus* and *O. uncinatus*), this structure was found in the vast majority of the nocturnal species (8 species of 9, with *S (Sc*.*) flavicornis* as the exception). The 44 species and 8 tribes of dung beetles examined in this study revealed an assortment of these morphological traits, independent of their taxonomic classification at tribe level; no <12 combinations of ecological (diurnal/crepuscular/nocturnal) morphological (presence/absence of a complete canthus and faceted/smooth cornea) and behavioral (rollers/tunnelers) features (Figures 1, 2, 3) were present within our samples (see Supporting Information).

#### 3.2.1 Diurnal species

While all 27 diurnal species had a faceted cornea, the presence or absence of a complete canthus varied within and between the tribes and nesting behaviors (Supporting Information). The eyes of the three species in the tribe Gymnopleurini, the four species in the tribe Sisyphyni, and five species in the tribe Onthophagini *lack* a complete canthus (e.g., Figure 1b,d) (Supporting Information), while the eye of the other four diurnal Onthophagini species is completely divided into a dorsal and a ventral part by the canthus (e.g., Figure 1c). Four of the five species in the tribe Oniticellini and all six diurnal Scarabaeini species (e.g., Figure 1a) also have an eye completely divided into a dorsal and a ventral part, while the complete canthus is absent from the eye of the *Afrodrepanus impressicollis*. See Supporting Information for a full list of species and eye type.

#### 3.2.2 Crepuscular species

The nine crepuscular species showed all possible combinations of morphological eye traits considered (presence or absence of a complete canthus, and faceted or smooth cornea surface) with the exception of the absence of a canthus combined with smooth cornea (Supporting Information). In addition, both rollers and tunnelers were found to be crepuscular. The presence of a complete canthus gives the tunneler *Onitis uncinatus* an eye completely divided into a dorsal and a ventral part with a smooth cornea (Figure 2c). The same combination, smooth eye and complete canthus, is observed in *Scarabaeus zambesianus* (Figure 2d). Six of the seven crepuscular species in the tribe Onthophagini have an eye characterized by the absence of a complete canthus, with a faceted cornea (e.g., Figure 2a; Supporting Information). The cornea is also facetted in *Onthophagus verticalis* (Onthophagini) and the canthus is complete (Figure 2b).

#### 3.2.3 Nocturnal species

The eight nocturnal species possess eyes with or without a complete canthus; but the cornea is *smooth*, with *S*. (*Sc*.) *flavicornis* as the exception (Figure 3d). Nocturnal species were found to be either rollers or tunnelers. The roller *Chalconotus convexus* has an eye characterized by the absence of a complete canthus and a smooth cornea. This eye structure holds true also for the five tunneler species in the tribe Coprini. See Supporting Information for a full list of species. The *presence* of a complete canthus separates the dorsal and ventral eye of both *Escarabaeus satyrus* and *S*. (*Sc*.) *flavicornis* (Figure 3a,d). The eyes of these very differently sized (see Table 2) roller species have a *smooth* or *facetted* cornea, respectively.

### 3.3 Comparison of body size and eye size between the species

**FIGURE 4:**
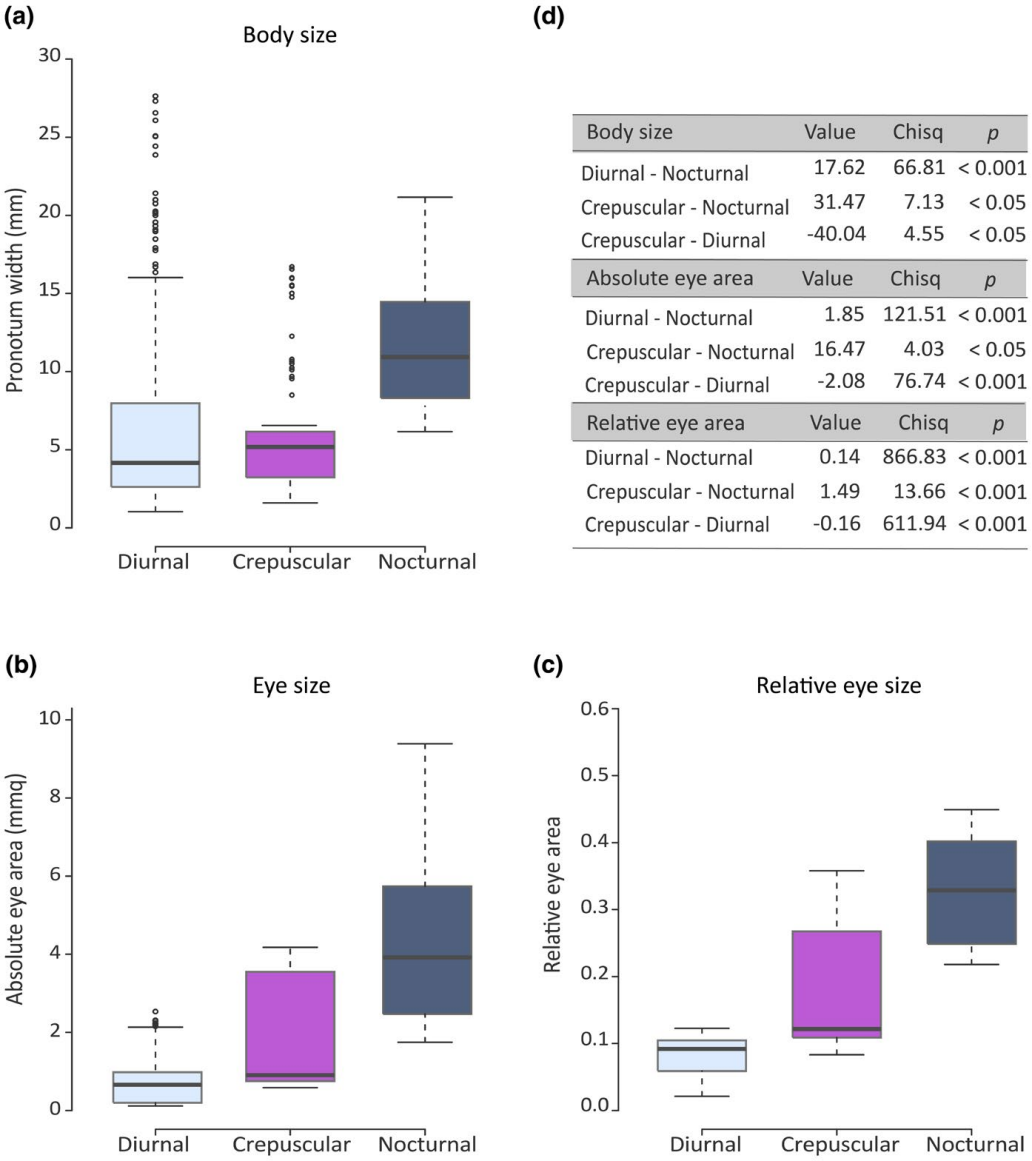
Body size (a), measured as the maximum width of the pronotum, of the 44 dung beetle species considered in the study. Absolute eye area (b) and relative eye size (c) of the 19 species which cover 12 combinations of morphological (*presence*/*absence* of the canthus and *facetted*/*smooth* cornea), ecological (*rollers*/*tunnelers*), and behavioral (*diurnal*/*crepuscular*/*nocturnal*) differences. Pairwise differences (d) in body size (44 species), absolute eye area (19 species), and relative eye area (19 species), and diel activity periods, were calculated with post hoc interaction analysis using the Chi‐squared test and correcting the *p*‐values according to the Holm method

Among our sample of 44 study species, we found that the nocturnal species are significantly larger (mean ± SD: 10.71 ± 3.94 mm, *n* = 79) compared to the crepuscular (mean ± SD: 5.38 ± 4.83 mm, *n* = 88) and diurnal species (mean ± SD: 6.59 ± 6.21 mm, *n* = 268), which also differ significantly in size from each other (Figure 4a,d). We further found that the rollers were significantly larger (mean ± SD: 11.91 ± 6.29 mm) compared to the tunneling species (mean ± SD: 4.39 ± 2.76 mm) (roller–tunneler: value = −6.96; Chisq = 74.42; *p* < .001).

To define differences in absolute eye area and relative eye area (i.e., the ratio between eye area and body size) among diurnal, crepuscular, and nocturnal species, we next defined the eye area of 19 of the species collected and measured. From this analysis, we found that the absolute eye area of the nocturnal species (mean ± SD: 4.42 ± 2.39 mmq, *n* = 48) was significantly greater than the eye area of the crepuscular (mean ± SD: 1.82 ± 1.80, *n* = 45) and the diurnal (mean ± SD: 0.84 ± 0.75, *n* = 77) species. Absolute eye area was also greater in the crepuscular species than in the diurnal species (Figure 4b,d). Similarly to absolute eye area, relative eye size in the nocturnal species (mean ± SD: 0.35 ± 0.06, *n* = 48) was significantly greater, followed by the crepuscular (mean ± SD: 0.19 ± 0.10, *n* = 45), and then the diurnal (mean ± SD: 0.08 ± 0.03, *n* = 77) species. Not unexpectedly, relative eye size was also greater in the crepuscular species than in the diurnal species (Figure 4c,d).

## 4 DISCUSSION

### 4.1 Diel activity period of dung beetle species

We found that the diel activity periods of our 44 dung beetle study species are so distinct that significant differences in activity could be defined, not only between but also *within* the different diurnal, crepuscular, and nocturnal guilds. This well‐defined “work shift schedule” of dung beetles appears to be an impressive example of temporal niche differentiation. However, visual or thermal physiology also offers alternative explanations for the patterns found.

Independent of tribe, genus, and nesting strategy, the diurnal species differ in their peaks of activity, as well as in their patterns of activity (unimodal or bimodal). For example, *Scarabaeus* (*Scarabaeolus*) *carniphilus* was mostly active at midmorning, while its congeneric *S*. (*Sc*.) *damarensis* reached a clear, second peak of activity midafternoon. Both species were found on Stonehenge farm (Tables 1 and 2). Similarly, unimodal or bimodal peaks of activity were adopted by the Oniticellini tunneler species collected on Pullen farm, where *Afrodrepanus impressicollis* and *Liatongus militaris* reached their peak of activity in midafternoon (Table 1), while *Drepanocerus kirbyi* and *Euoniticellus intermedius* were instead active midmorning and midafternoon (Table 2). This allows for temporal partitioning of the dung habitat, especially for the ball‐rolling species, which leave the dung pat shortly after they have formed a ball. Also the dim light–active species have distinct and well‐defined peaks of activity distributed among their crepuscular and nocturnal times (Table 2). Differences in activity are even visible in the short crepuscular times, where, for instance, the tunneler *Onthophagus variegatus* (Figure 2a) reaches its dusk peak of activity 30 min earlier than its congeneric *O. vinctus*. The crepuscular tunneler *Onitis uncinatus* (Figure 2c), as reported by Caveney et al. (1995), and *Onthophagus flavimargo*, and the crepuscular roller *Scarabaeus zambesianius* (Figure 2d) have a less acute peak of activity than the other crepuscular species, with some individuals extending their activity to the night (Table 2). This extreme variety in diel activity period between sympatric species is independent of their taxonomic classification and nesting behavior.

Dusk presents challenging light conditions for most visually driven behaviors of any active animal (Kelber et al., 2006; Malmqvist et al., 2018). Nevertheless, it is the most sharply defined activity period we observed, representing a visual watershed that only *K. signatus* of the diurnal species examined was able to cross. This narrow time slot presumably offers warm temperatures along with predator evasion due to the rapidly changing light intensity (Malmqvist et al., 2018). The lower number of species and individuals (Table 2) active at similar light levels during dawn is presumably the result of low morning temperatures inhibiting flight (Zhang et al., 2010). Once the sun has fully risen, however, light intensity during the day changes very little (Lythgoe, 1979). While most of our model species show a clear drop‐in activity at midday (which coincides with the warmest hours of the day), the small tunneling species rather seem to take advantage of the heat (Table 1). For them, the high midday temperature is possibly less challenging because they do not make contact with the hot soil that can cause rolling species to stilt on their ball (Smolka et al., 2012). Given that all tunneling species will remain in the dung pat or underneath it for at least several days after their arrival, competition is expected to ensue in the dung and the soil below it, for food and space. This suggests that the clearly defined foraging periods we noted might be more closely linked to the beetles’ physiology and reflected in the their body size and eye structure, rather than avoidance of competition for dung or space per se.

### 4.2 Body and eye size is bigger in dim light–active beetles

The temporal separation not only structures the communities of dung beetles (Caveney et al., 1995; Feer & Pincebourde, 2005; Kamiński et al., 2015), but it also reflected their morphology (Tocco et al., 2019). An analysis of body size in our 44 model species of dung beetles clearly shows that nocturnal beetles have a significantly larger body than crepuscular and diurnal species (Figure 4a). This is not a surprise, as nocturnal activity is often associated with larger body size in beetles (Hernández et al., 2011; Nichols et al., 2013), moths (Tammaru et al., 2018), and several other orders of insects (Guevara & Avilés, 2013). This larger body volume has been suggested to help nocturnal beetles to fly at the low nighttime temperatures (Bartholomew & Heinrich, 1978; Lobo & Cuesta, 2021; Verdú et al., 2006); allow stingless bees to start foraging in the cooler early morning (Pereboom & Biesmeijer, 2003) and forager leaf‐cutting ants to perform well into the night (Wetterer, 1990). However, in contrast to Hernández et al. (2011), we also found that the range of body sizes was narrower in our sample of nocturnal species than in our diurnal sample. This opposing trend in the range body size of diurnal and nocturnal dung beetles noted between the two studies could be an effect of our species selection. However, given that our analysis reflects the total abundance of the species selected, we feel that our conclusion supports the anecdotal observation that nocturnal species are generally large. Our results also draw attention to the fact that, in a dung beetle community, not only the relative eye size (Figure 4d) but also the absolute eye size (Figure 4c) of nocturnal species are significantly greater, followed by crepuscular and then diurnal species. In dim light, active beetle species' eyes are “photon starved.” As light intensity falls, the number of photons available for the photoreceptors in the eye decreases so that the ability to detect contrast (signal‐to‐noise ratio) is reduced and the quality of the image compromised (Land & Nilsson, 2012). As a larger eye can accommodate bigger photoreceptors and lenses it supports a higher light sensitivity, and this is one way to compensate for photon shortage (Land & Nilsson, 2012; Warrant & Dacke, 2011). Not surprisingly, an enlargement of the eye and its optical elements can be found in several nocturnal animals, including owls (Lisney et al., 2012), teleost reef fish (Schmitz & Wainwright, 2011), halictid bees (Greiner et al., 2004), lemurs (Peichl et al., 2019), and dung beetles (McIntyre & Caveney, 1998; Tocco et al., 2019; Warrant & Dacke, 2011). Conversely, the relatively smaller eye size in the diurnal species can also be considered adaptive. Niven et al. (2007) found that flies eyes reflect a trade‐off between performance and the energetic costs of vision. They showed that large blowfly eyes were more costly to run than the smaller eyes of drosophila, which coded the same quantity of information using one‐tenths of the energy of the larger species. Precisely defining the diel activity period of a wide range of dung beetle species allowed us to conclude that the temporal partitioning strategy, but not nesting behavior or taxonomic status, is the main driver of eye morphology. A broader analysis of other beetle communities beyond the three we studied could potentially confirm if the phylogenetic signal is completely absent, suggesting that diel flight times have arisen more than once during the course of evolution. But that would still not answer why this should be so.

### 4.3 Corneal structure is driven by diel activity

While the eyes of the 27 diurnal species of this study had an obviously faceted cornea (Figure 1), some crepuscular (2 of 9 species) and the large majority of the nocturnal (7 of 8 species) species had a smooth, glassy cornea without any visible external borders between the small optical units of the eye (Figure 3). The smooth corneal surface that gives a glassy appearance occurs when the curvature of the single facets and the curvature of the total cornea match each other (Caveney & McIntyre, 1981). In insects, a totally flat corneal surface is relatively rare, but can be found in hissing cockroaches (Mishra & Meyer‐Rochow, 2008), halictid bees (Greiner et al., 2004), and several families of beetles (Blagodatski et al., 2015; Caveney & McIntyre, 1981; Gokan et al., 1998). In a former study that covers the corneal structure of 45 different species of scarab beetles, including six crepuscular and seven nocturnal dung beetle species (one of them in common with our study), Caveney and McIntyre (1981) suggested that a *smooth* cornea is one of the many morphological light adaptations used by some crepuscular and nocturnal scarabs to overcome visual limitations in dim light conditions, but incomplete activity records of their study species made it difficult to draw any firm conclusions. In a similar way, Gokan et al. (1998), studying the eye morphology of six Japanese stag beetles, hypothesized that the species with more convexly curved facets (*Aesalus asiaticus* Lewis) was the only truly diurnal species within that sample. Again, the lack of information on activity periods left this hypothesis unverified. Our results clearly show that the corneal structure (faceted/smooth) is strongly correlated with the diel activity period of the dung beetle species rather than to taxonomy or nesting behavior.

While our results do not at this time explain why the eye of the nocturnal *S*. (*Sc*.) *flavicornis* has a *facetted* cornea (Figure 3d), we can exclude the taxonomy, nesting behavior, or body size as the reason for this anomaly. Future investigations into the internal structures of smooth and faceted dung beetle eyes are currently being undertaken, aiming to reveal the optical basis of this dim light adaptation.

### 4.4 The enigmatic role of the canthus

While all members of the Scarabaeini in our sample (and in general) are characterized by the presence of a complete ocular canthus, and the Sisyphini and Coprini species by the absence of the same, the presence/absence of this structure does not necessarily provide a stable taxonomic classification within the subfamily Scarabaeinae. In the tribes Onthophagini and Oniticellini, for example, only 5 of the 16 and 4 of the 5 species included in this study, respectively, showed a complete ocular canthus (Supporting Information). The giant dung beetles of the genus *Heliocopris* are, indeed, one of the few cases where differences in the shape of the ocular canthus between species are described and used in dung beetle dichotomous keys (Pokorny et al., 2009).

It has been suggested that the ocular canthus protects the eyes of dung beetles both from abrasion while digging and from damage resulting from collisions with objects during flight (Scholtz & Davies, 2009). While this might indeed be part of its function, it is not present in all dung beetles that burrow into the soil. In our study sample of 44 species of tunnelers and rollers, a complete ocular canthus was only present in 19 of these (Supporting Information). Neither does soil type seem to be a determining factor. Among the Stonehenge and Pullen farm assemblages, that is, beetles active exactly in the same soil type, 13 of 26 species and 6 of 18 species, respectively, had a complete ocular canthus (Supporting Information). In summary, we did not find any correlation between this structure and nesting behavior (roller or tunneler) or time of activity (diurnal or nocturnal). At this time, we can only, once again (Byrne & Dacke, 2011), conclude that the function of the ocular canthus in dung beetles remains enigmatic.

## 5 CONCLUSIONS

An accurate knowledge of diel activity period is essential for a full understanding of morphological light adaptations that support a nocturnal lifestyle. In dung beetles, we found that nocturnal species are larger than crepuscular and diurnal species, and that absolute and relative eye size is greatest in nocturnal species, followed by crepuscular, and then diurnal species. A more surprising finding was that corneal structure was influenced by the activity period of the species; flat in the nocturnal species and highly curved in the diurnal ones. Interestingly, this functional adaptation for dim light vision has largely escaped the attention of visual scientists. The role of the canthus—the cuticular structure that partially or completely divides the dung beetle eye into dorsal and ventral parts—remains a mystery, but the large number of species investigated in this study still allowed us to reject any correlation with nesting behavior or time of activity.

## CONFLICT OF INTEREST

The authors declare no conflicts of interest.

## AUTHOR CONTRIBUTIONS

**Claudia Tocco:** Conceptualization (equal); Data curation (equal); Formal analysis (equal); Methodology (equal); Visualization (equal); Writing‐original draft (equal). **Marie Dacke:** Conceptualization (equal); Funding acquisition (equal); Methodology (equal); Supervision (equal); Validation (equal); Writing‐review & editing (equal). **Marcus Byrne:** Conceptualization (equal); Data curation (equal); Methodology (equal); Supervision (equal); Validation (equal); Writing‐review & editing (equal).

## Supporting information

Table S1–S2

## Data Availability

All data supporting reported results can be found in the manuscript and Supporting Information.
