# Supplementary material for: The finely defined shift work schedule of dung beetles and their eye morphology
Source: Ecol Evol. 2021 Oct 27;11(22):15947–60. doi: 10.1002/ece3.8264 (PMC8601928; doi:10.1002/ece3.8264)
Supplement: Supplementary file 1 — Table S1–S2 [file ECE3-11-15947-s001.pdf]

Supporting Information

Table S1. List of tribes and species sampled in Stonehenge game farm (26°28′14.0"S 24°20′30.8"E). Sampling session: (A) 14th November 2016, (B) 18th November 2018, (C) 29th January 2017, (D) 15th November 2017, and (E) 29th January 2018. Activity period: D = diurnal, C = crepuscular, and N = nocturnal. Cornea: F = facetted and S = smooth Canthus: P = complete canthus present, and A = complete canthus absent. Nesting behaviour: T = tunneler, and R = roller.

		Stonehenge game farm										
		**Sampling session**	A	B	C	D	E	Total	Activity period	Cornea	Canthus	Nesting behaviour
		**Number of sampling times**	20	20	20	18	17					
		**Number of traps**	5	5	5	5	5					
		**Species richness**	46	33	46	43	35	52				
		**Number of individuals**	2811	881	3600	2999	645	10936				
Species sampled in numbers exceeding 100 individuals	Coprini	Copris cassius Péringuey	35	7	70	44	4	160	N	S	A	T
		Copris elphenor Felsche	44	12	37	5	7	105	N	S	A	T
		Metacatharsius latifrons (Harold)	46	13	26	115	2	202	N	S	A	T
		Metacatharsius opacus Waterhouse	3	0	14	69	22	108	N	S	A	T
	Gymnopleurini	*Allogymnopleurus splendidus* (Bertoloni)	238	317	223	51	3	832	D	F	A	R
		Gymnopleurus aenescens Wiedemann	50	8	27	17	0	102	D	F	A	R
	Oniticellini	*Euoniticellus intermidius* (Reiche)	170	12	2	24	3	211	D	F	P	T
	Onthophagini	*Caccobius cavatus* D`Orbigny	32	43	15	6	31	127	C	F	A	T
		Kurtops quadraticeps (Harold)	167	43	205	84	15	514	D	F	P	T
		Kurtops signatus (Fahraeus)	289	98	1482	393	133	2395	D	F	P	T
		Onthophagus flavimargo D'Orbigny	17	0	29	71	5	122	C	F	A	T
		Onthophagus flavolimbatus Klug	44	4	105	54	12	219	D	F	A	T
		Onthophagus granulifer Harold	35	18	28	59	23	163	C	F	A	T
		Onthophagus pallidipennis Fåhr	77	12	13	36	31	169	D	F	A	T
		Onthophagus variegatus Fabricius	830	137	777	710	66	2520	C	F	A	T
		Onthophagus verticalis Fahraeus	2	0	147	6	5	160	C	F	P	T
		Onthophagus vinctus Erichson	30	0	49	81	3	163	C	F	A	T
		Onthophagus sp_w04	78	6	64	167	23	338	D	F	P	T
		Proagoderus sapphirinus Fåhr	46	27	18	112	2	205	D	F	P	T
	Scarabaeini	*Escarabaeus satyrus* (Boheman)	85	9	15	41	15	165	N	S	P	R
		Kheper lamarcki (Mac Leay)	99	37	49	251	28	464	D	F	P	R
		Pachylomera femoralis Kirby	73	18	17	39	40	187	D	F	P	R
		Scarabaeus (Scarabaeolus) carniphilus Davis	36	8	18	43	3	108	D	F	P	R
		Scarabaeus (Scarabaeolus) damarensis Janssens	27	1	1	237	0	266	D	F	P	R
		Scarabaeus (Scarabeolus) flavicornis Boheman	17	3	29	41	11	101	N	F	P	R
		Scarabaeus zambesianus Péringuey	91	14	21	123	108	357	C	S	P	R

**Species sampled in numbers not exceeding 100 individuals**	Coprini	Catharsius sp_w01	4	5	26	4	3	42
		Copris sp_w02	1	6	0	0	0	7
		Metacatharsius sp_w02	3	0	22	15	2	42
	Dichotomiini	*Heliocopris japetus* Klug	0	0	3	0	9	12
		Pedaria sp_w01	3	3	4	4	1	15
		Pedaria sp_w02	0	0	6	0	0	6
		Pedaria sp_w03	0	0	0	1	0	1
		Sarophorus sp_w01	0	0	0	1	0	1
	Odontolomini	Odontoloma sp_w01	2	0	3	0	0	5
	Oniticellini	Drepanellus laticollis	15	7	11	1	2	36
		Drepanocerus patrizii (Boucomont)	1	2	4	1	0	8
		Liatongus militaris (Castelnau)	1	3	7	1	0	12
		Oniticellus pictus (Péringuey)	0	1	0	0	0	1
	Onitini	Onitis sp_w01	1	0	7	0	0	8
	Onthophagini	*Caccobius ferrugineus* Fahraeus	29	0	7	5	4	45
		Caccobius nigritulus Klug	4	0	1	17	12	34
		Cleptocaccobius viridicollis (D'Orbigny)	21	0	4	18	0	43
		Digitonthophagus gazella (Fabricius)	7	0	1	0	8	16
		Onthophagus sp_w09	24	3	2	23	1	53
		Onthophagus sp_w10	12	1	1	17	0	31
		Phalops wittei (Harold)	2	0	0	1	0	3
	Scarabaeini	*Kheper prodigiosus* (Erichson)	3	2	4	5	7	21
		Scarabaeus (Scarabeolus) afronitidus Davis and Deschodt	0	0	0	1	0	1
		Scarabaeus (Scarabeolus) anderseni Waterhouse	1	0	3	5	0	9
	Sisyphini	*Neosishypus calcaratus* Klug	6	1	2	0	1	10
		Neosisyphus rubrus (Paschalidis)	10	0	1	0	0	11

Table S2. List of tribes and species sampled in Pullen nature reserve (25°34′01.6"S 31°10′42.5"E). Sampling session: (A) 26th February 2019, (B) 25th March 2019, and (C) 10th November 2019. Activity period: D = diurnal, C = crepuscular, and N = nocturnal. Cornea: F = facetted and S = smooth Canthus: P = complete canthus present, and A = complete canthus absent. Nesting behaviour: T = tunneler, and R = roller.

		**Pullen nature reserve**								
		**Sampling day**	A	B	C		**Activity period**	**Cornea**	**Canthus**	**Nesting behaviour**
		**Number of sampling times**	10	11	10					
		**Number of traps**	10	10	10					
		**Species richness**	34	31	30	35				
		**Number of individuals**	2037	1207	3576	6820				
**Species sampled in numbers exceeding 100 individuals**	Coprini	*Copris mesacanthus transvaalensis* Nguyen-phung	49	27	53	129	N	S	A	T
	Deltochilini	*Chalconotus convexus* Boheman	138	25	70	233	N	S	A	R
	Gymnopleurini	*Garreta unicolor* (Fahraeus)	320	18	633	971	D	F	A	R
	Oniticellini	*Afrodrepanus impressicollis* (Boheman)	194	50	577	821	D	F	A	T
		Drepanellus laticollis (Fahraeus)	37	57	149	243	D	F	P	T
		Drepanocerus Kirbyi Kirby	69	25	29	123	D	F	P	T
		Euoniticellus intermedius (Reiche)	65	59	12	136	D	F	P	T
		Liatongus militaris (Laporte)	38	35	35	108	D	F	P	T
	Onthophagini	*Onthophagus asperulus* D'Orbigny	124	197	91	412	D	F	A	T
		Onthophagus cribripennis D'Orbigny	41	59	314	414	D	F	A	T
		Onthophagus fimetarius Roth	11	3	142	156	C	F	A	T
		Onthophagus sugillatus Klug	21	15	337	373	D	F	A	T
	Scarabaeini	*Kheper nigroaeneus* (Boheman)	44	49	70	163	D	F	P	R
		Scarabaeus rusticus (Boheman)	61	46	165	272	D	F	P	R
	Sisyphini	*Sisyphus fasciculatus* Boheman	353	311	275	939	D	F	A	R
		Sisyphus manni Montreuil	70	44	4	118	D	F	A	R
		Sisyphus seminulum Gerstaecker	199	52	373	624	D	F	A	R
		Sisyphus sordidus Boheman	24	48	155	227	D	F	A	R
**Species sampled in numbers not exceeding 100 individuals**	Coprini	*Catharsius sesostris* Waterhouse	7	8	11	26				
	Dichotomiini	*Sarophorus sp_pul01*	4	0	7	11				
	Gymnopleurini	*Garetta sp_pul01*	2	0	6	8				
	Oniticellini	*Drepanocerus orientalis* Krikken	16	1	0	17				
		Eodrepanus bechynei	3	1	0	4				
		Eodrepanus parallelus (Raffray)	1	0	0	1				
		Tiniocellus eurypygus transdrakensbergensis Branco	73	2	12	87				
	Onitini	*Onitis alexis* Klug	4	3	4	11				
		Onitis caffer Boheman	0	4	0	4				
		Onitis fulgidus Klug	9	3	6	18				
		Onitis picticollis Boheman	4	4	1	9				
	Onthophagini	*Onthophagus pallidipennis* Fåhr	22	11	11	44				
		Onthophagus flavolimbatus Klug	5	0	0	5				

Scarabaeini	*Scarabaeus caffer* Boheman	3	23	22	48
Sisyphyni	*Neosishypus spinipes* Thunberg	4	2	7	13
	Neosisyphus rubrus (Paschalidis)	13	4	3	20
	Sishyphus sp_Pul02	9	21	2	32

* *Onitis uncinatus* Klug [tribe Onitini] was sampled near the town of Bela Bela, Limpopo Province (24°46′04.0"S 27°56′37.3"E) on the 28th November 2019 (16 individuals) and 15th February 2020 (7 individuals). This species was used as a representative of a *crepuscular* and *tunneler* species with an eye with a *complete canthus* and a *smooth cornea*.
